# Relapse Associated with Active Disease Caused by Beijing Strain of *Mycobacterium tuberculosis*^1^

**DOI:** 10.3201/eid1507.081253

**Published:** 2009-07

**Authors:** William J. Burman, Erin E. Bliven, Lauren Cowan, Lorna Bozeman, Payam Nahid, Lois Diem, Andrew Vernon, for the Tuberculosis Trials Consortium

**Affiliations:** Denver Public Health, Denver Colorado, USA (W.J. Burman); University of Colorado at Denver Health Sciences Center, Denver (W.J. Burman); Centers for Disease Control and Prevention, Atlanta, Georgia, USA (E.E. Bliven, L. Cowan, L. Bozeman, L. Diem, A. Vernon); The University of California, San Francisco, California, USA (P. Nahid)

**Keywords:** Beijing strain, Mycobacterium tuberculosis, tuberculosis and other mycobacteria, genotyping, relapse, risk factor, race and ethnicity, podcast, research

## Abstract

The role of microbial factors in outcomes of tuberculosis treatment has not been well studied. We performed a case–control study to evaluate the association between a Beijing strain and tuberculosis treatment outcomes. Isolates from patients with culture-positive treatment failure (n = 8) or relapse (n = 54) were compared with isolates from randomly selected controls (n = 296) by using spoligotyping. Patients with Beijing strains had a higher risk for relapse (odds ratio [OR] 2.0, 95% confidence interval [CI] 1.0–4.0, p = 0.04) but not for treatment failure. Adjustment for factors previously associated with relapse had little effect on the association between Beijing strains and relapse. Beijing strains were strongly associated with relapse among Asian–Pacific Islanders (OR 11, 95% CI 1.1–108, p = 0.04). Active disease caused by a Beijing strain was associated with increased risk for relapse, particularly among Asian–Pacific Islanders.

## Abstract 2

Risk for relapse was higher among persons of Asian–Pacific Islander descent.

Approximately 2%–5% of patients with tuberculosis (TB) treated with contemporary short-course treatment either fail to respond to therapy or recurrent TB can develop in these patients after they complete therapy, despite assurance of adherence through supervised treatment (*1*,*2*). In settings with a high prevalence of disease, a substantial percentage of recurrent cases are caused by reinfection with another strain of *Mycobacterium tuberculosis* (*3*,*4*). However, reinfection is an uncommon cause of recurrent disease in settings in which the prevalence of active TB is low (*5*). Several studies have evaluated risk factors for recurrent TB, which are severity of the radiographic manifestations of disease (presence of cavitation [*1*,*6*–*8*], extent of pulmonary involvement [*1*,*6*,*8*], or the presence of silicosis [*9*]), microbial load at diagnosis (*7*), and 2-month sputum culture positivity as an indicator of the early response to therapy (*1*,*6*,*7*).

Whether aspects of the infecting strain of *M*. *tuberculosis* might affect treatment outcomes has not been well studied. Studies early in the time of chemotherapy found that *M*. *tuberculosis* strains from patients who responded well to isoniazid monotherapy seemed to be somewhat less virulent in a guinea pig model of active TB (*10*). However, this line of investigation was not pursued in the context of response to multidrug therapy. DNA fingerprinting techniques enable classification of *M*. *tuberculosis* isolates into genotype families. The Beijing genotype family has received considerable attention because of its association with drug resistance (*11*,*12*). Furthermore, the Beijing family may be rapidly spreading in some areas (*13*–*15*). Studies from Vietnam (*16*) and Singapore (*17*) showed that active disease caused by a Beijing strain was associated with an increased risk for recurrent TB after completion of treatment.

Recent studies have shown associations among *M*. *tuberculosis* strains, geographic regions, and human populations, which suggest that specific strains of *M*. *tuberculosis* coevolved with human subpopulations (*18*–*20*). However, with increased population mobility, there has been greater mixing of *M*. *tuberculosis* strains and human subpopulations. The interaction between these factors (the bacillary strain and the race/ethnic background of the patient), may affect response to TB treatment (*21*). We used isolates from a large multicenter clinical trial (Tuberculosis Trials Consortium [TBTC] Study 22) (*1*) to evaluate the association between active disease caused by a Beijing strain and TB treatment outcomes. We also explored whether this association was affected by the race/ethnicity of the patient.

## Methods

### Study Population

TBTC Study 22 was a randomized trial comparing once-a-week rifapentine plus isoniazid with twice-a-week rifampin plus isoniazid during the last 4 months of short-course treatment for drug-susceptible pulmonary TB (*1*,*22*). Adults were enrolled from sites in the United States and Canada from 1995 through 1998. Before enrollment, all patients had completed an initial 2 months of treatment with isoniazid, rifampin, pyrazinamide, and ethambutol (or streptomycin). All TB treatment was supervised (directly observed therapy).

Sputum cultures were obtained monthly during treatment. Failure was defined as a positive culture >4 months of treatment. Relapse was defined as a positive culture during the 2-year follow-up after completion of therapy. Paired isolates from the time of enrollment and the time of suspected treatment failure or relapse underwent insertion sequence (IS) *6110* fingerprinting (*1*,*23*), and an endpoint review committee determined whether the positive culture was caused by cross-contamination, relapse, or reinfection. TBTC Study 22 was reviewed and approved by the Institutional Review Boards of CDC and participating sites. The present analysis of isolates from that study was reviewed and found to be research that did not include human patients.

### Selection of Case-Patients and Controls

We evaluated the association between active disease caused by a Beijing strain and TB treatment outcomes by using a nested case–control study of isolates from participants in TBTC Study 22. Because it is likely that the risk factors for treatment failure and relapse are different among persons with HIV co-infection, we limited this case–control analysis to HIV-negative participants (n = 1,004). Case-patients were participants who adhered to study therapy and had culture-positive treatment failure or relapse caused by the initial infecting strain of *M*. *tuberculosis*. Controls were selected through simple random sampling of participants who completed treatment and had 2 years of follow-up (3, 6, 9, 12, 18, and 24 months after treatment completion) with no clinical or microbiologic evidence of treatment failure or relapse. Cases and controls were not matched for any demographic, clinical, or radiographic characteristics.

### Laboratory Methods

Isolates from cases and controls underwent spoligotyping (*24*) as modified by Cowan et al. (*25*). Beijing strains were defined as isolates with a spoligotype showing the absence of spacers 1–34 and presence of at least 3 spacers among spacers 35–43 (*26*,*27*). To evaluate possible associations between *M*. *tuberculosis* lineages, as defined by large sequence polymorphisms and treatment outcomes, isolates were assigned by spoligotype pattern to previously described lineages (*20*,*21*).

### Statistical Analyses

The number of cases was determined from the parent clinical trial; 4 controls were randomly selected for each case. Race/ethnicity was determined by site staff who used categories defined in the United States TB surveillance system (*28*).

The definitions and primary objective of this case–control analysis were formulated before data analysis. The initial analysis evaluated the association between active disease caused by a Beijing strain and treatment failure or relapse. To enable comparisons with previous studies (*16*,*17*), we then analyzed relapse alone as the outcome. We subsequently analyzed the relationships between race/ethnicity, active disease caused by a Beijing strain, and treatment outcomes. Associations between active disease caused by a Beijing strain and baseline characteristics and treatment outcomes were evaluated by using χ^2^ analysis. Associations with treatment outcomes were adjusted in multivariate logistic regression models for factors previously associated with failure and relapse. These factors were white race, being underweight, pulmonary cavitation, bilateral pulmonary involvement, and 2-month sputum culture positivity (*1*). Because having been randomly assigned rifapentine as treatment was associated with an increased risk for relapse in univariate analyses of TBTC Study 22 (*1*), we conducted a secondary analysis in which treatment assignment was forced into multivariate models of risk for poor treatment outcomes.

## Results

Of the 1,004 HIV-negative participants in TBTC Study 22, there were 8 instances of treatment failure and 61 cases of relapse. Of these 69 cases of adverse TB treatment outcomes, isolates from 64 patients were successfully genotyped (Figure). Of the 930 study participants who did not experience treatment failure or relapse, 76 did not complete therapy and 172 did not complete follow-up. Of the remaining 687 patients, 296 (43%) were randomly selected as controls for this analysis; their 296 baseline isolates were successfully genotyped (Figure).

**Figure:**
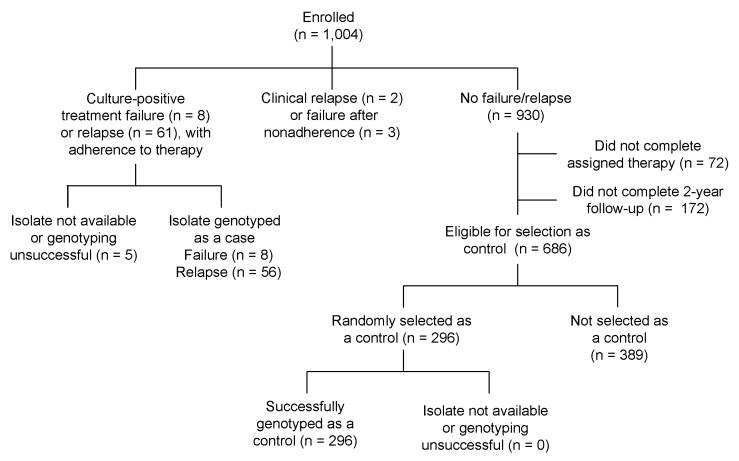
Selection of case-patients and controls, Tuberculosis Trials Consortium Study 22. The Tuberculosis Trials Consortium Study enrolled patients during 1995–1998. Participants in the case–control study were selected from among 1,004 HIV-infected participants.

Demographic and clinical characteristics of cases and controls are provided in Table 1. As in the entire study cohort, white race, being underweight, pulmonary cavitation, bilateral pulmonary involvement, and 2-month sputum smear and culture positivity were associated with an increased risk for treatment failure and relapse among the participants of this case–control analysis (Table 1). Patients with active disease caused by a Beijing strain had an increased risk for treatment failure or relapse (15 [23%] of 64 cases vs. 42 [14%] of 296 controls; odds ratio [OR] 1.9, 95% confidence interval [CI] 0.9–3.6, p = 0.07). When analyzed as separate endpoints (Table 2), treatment failure was not associated with active disease caused by a Beijing strain (OR 0.9, 95% CI 0.1–7.2, p = 1.00), but relapse was significantly associated (OR 2.0, 95% CI 1.0–4.0, p = 0.04). Active disease caused by the Indo-Oceanic lineage was associated with a lower risk for treatment failure or relapse, although the small number of isolates from that lineage (n = 25) and the lack of any cases of treatment failure or relapse among patients with active disease caused by this lineage precluded further evaluation of this association.

**Table 1:** Characteristics of case-patients and controls, Tuberculosis Trials Consortium Study 22*

Characteristic	Case-patients (treatment failure or relapse), n = 64†	Controls (patients cured), n = 296†	Odds ratio (95% confidence interval)	p value
Demographic				
Age, y, mean (SD)	42 (13)	44 (14)	1.0 (0.97–1.01)	0.51
Men	54 (84)	214 (72)	2.1 (1.0–4.3)	0.05
Treatment				
Rifapentine, 1×/wk	40 (63)	151 (51)	1.6 (0.9–2.8)	0.10
Rifampin, 2×/wk	24 (37)	145 (49)		
Ethnic origin				
Non-Hispanic white	22 (34)	43 (15)	3.1 (1.7–5.7)	0.0002
Non-Hispanic black	25 (39)	127 (43)	0.9 (0.5–1.5)	0.57
Hispanic	9 (14)	71 (24)	0.5 (0.2–1.1)	0.08
Asian–Pacific Islander	6 (9)	45 (15)	0.6 (0.2–1.4)	0.23
Native American	2 (3)	10 (3)	0.9 (0.2–4.3)	0.92
Birthplace				
United States or Canada	48 (75)	200 (68)	1.4 (0.8–2.7)	0.24
Mexico	5 (8)	36 (12)	0.6 (0.2–1.6)	0.32
Europe	2 (3)	4 (1)	2.4 (0.4–13.1)	0.31
Southeast Asia	2 (3)	6 (2)	1.6 (0.3–7.9)	0.59
Western Pacific	4 (6)	33 (11)	0.5 (0.2–1.6)	0.24
Other	3 (5)	17 (6)	0.8 (0.2–2.8)	0.74
Baseline clinical features				
Fever	50/62 (81)	166/289 (57)	3.1 (1.6–6.0)	0.0007
Sweats	42/63 (67)	162/287 (56)	1.5 (0.9–2.7)	0.14
Cough	61 (95)	256/294 (87)	3.0 (0.9–10.1)	0.06
Underweight‡	38 (59)	82 (28)	3.8 (2.2–6.7)	<0.0001
Sputum smear positive	55 (86)	193/292 (66)	3.1 (1.5–6.6)	0.002
Baseline chest radiographic features				
Cavitation	54 (84)	146/287 (51)	5.2 (2.6–10.6)	<0.0001
Bilateral pulmonary involvement	50 (78)	155/293 (53)	3.2 (1.7–6.0)	0.0002
Two-month sputum analysis				
Smear positive	16 (25)	30/285 (11)	2.8 (1.4–5.6)	0.002
Culture positive	33 (54)	48/267 (18)	5.4 (3.0–9.7)	<0.0001
*Mycobacterium tuberculosis* lineage/family				
East Asian/Beijing	15 (23)	42 (14)	1.9 (0.9–3.6)	0.07
Euro-American	47 (73)	221 (75)	0.9 (0.5–1.7)	0.84
Indo-Oceanic	0	25 (8.5)	0.08 (0.01–1.4)	0.01
East African	1 (1.6)	3 (1.0)	1.6 (0.16–15.1)	0.54
Unclassified lineage	1 (1.6)	5 (1.7)	0.9 (0.11–8.04)	1.00

*The Tuberculosis Trials Consortium Study enrolled patients during 1995–1998. Participants in the case–control study were selected from among 1,004 HIV-infected participants. †Except for age, values are no. (%) or no. positive/no. tested (%). ‡Less than 10% below ideal bodyweight at diagnosis.

**Table 2:** Association between treatment failure or relapse and active disease caused by Beijing vs. other genotypes of *Mycobacterium tuberculosis*, Tuberculosis Trials Consortium Study 22*

Outcome	Disease caused by Beijing genotype, n = 57	Disease caused by other genotype, n = 303	Odds ratio (95% CI)	p value
Cure (n = 296)	42 (14)	254 (86)	1.9 (0.9–3.6)	0.07
Failure or relapse (n = 64)	15 (23)	49 (77)		
Failure (n = 8)	1 (13)	7 (88)	0.9 (0.1–6.3)	1.00
Relapse (n = 56)	14 (25)	42 (75)	2.0 (1.0–4.0)	0.04

*The Tuberculosis Trials Consortium Study enrolled patients during 1995–1998. Participants in the case–control study were selected from among 1,004 HIV-infected participants. CI, confidence interval.

Adjustment for the 5 clinical, radiographic, and microbiologic risk factors for treatment failure and relapse in the parent study had little effect on the OR of active disease being caused by a Beijing strain and relapse (adjusted OR 2.2, 95% CI 1.0–5.0, p = 0.05) (Table 3). Similarly, treatment assignment (rifapentine vs. rifampin) had little effect in a multivariate model into which this factor was forced (adjusted OR for active disease caused by Beijing strain 2.3; 95% CI 1.0–5.3, p = 0.04).

**Table 3:** Association between active disease caused by a Beijing genotype of *Mycobacterium tuberculosis* and relapse, adjusted for clinical risk factors, Tuberculosis Trials Consortium Study 22*

Characteristic	Univariate analysis			Multivariate analysis	
	Odds ratio (95% CI)	p value		Odds ratio (95% CI)	p value
Infected with Beijing genotype	2.0 (1.0–4.0)	0.05		2.2 (1.0–4.9)	0.07
Non-Hispanic white race/ethnicity	3.3 (1.7–6.1)	<0.01		3.0 (1.4–6.7)	<0.01
Underweight at tuberculosis diagnosis	4.7 (2.6–8.6)	<0.01		3.7 (1.8–7.2)	<0.01
Pulmonary cavitation	5.0 (2.4–10.7)	<0.01		3.2 (1.4–7.5)	0.01
Bilateral pulmonary disease	2.9 (1.5–5.7)	<0.01		1.8 (0.9–4.0)	0.12
Two-month sputum culture positivity	4.7 (2.6–8.7)	<0.01		2.4 (1.2–4.9)	0.01

*The Tuberculosis Trials Consortium Study enrolled patients during 1995–1998. Participants in the case–control study were selected from among 1,004 HIV-infected participants. CI, confidence interval.

We next evaluated the effect of race/ethnicity on the association between relapse and active disease caused by a Beijing strain (Table 4). Asian–Pacific Islanders were not at increased risk for relapse in the entire TBTC Study 22 cohort or in this nested case–control analysis (OR 0.6, 95% CI 0.2–1.4, p = 0.23). However, Asian–Pacific Islanders who had active disease caused by a Beijing strain were at increased risk for relapse (OR 11, 95% CI 1.0–108, p = 0.04) (Table 4).

**Table 4:** Association between active disease caused by a Beijing genotype of *Mycobacterium tuberculosis* and relapse, by race/ethnic background, Tuberculosis Trials Consortium Study 22*

Race/ethnicity	Patients with disease that relapsed		Relapse odds ratio (95% CI)	p value
	Infected with a Beijing strain, no. positive/no. tested (%)	Not infected with a Beijing strain, no. positive/no. tested (%)		
Non-Hispanic white (n = 63)	3/11 (27)	17/52 (33)	0.8 (0.2–3.3)	0.73
Non-Hispanic black (n = 148)	6/22 (27)	15/126 (12)	2.8 (0.9–8.2)	0.06
Hispanic (n = 79)	1/7 (14)	7/72 (9.7)	1.5 (0.2–15)	0.70
Asian–Pacific Islander (n = 50)	4/16 (25)	1/34 (2.9)	11 (1.1–108)	0.04

*The Tuberculosis Trials Consortium Study enrolled patients during 1995–1998. Participants in the case–control study were selected from among 1,004 HIV-infected participants. Participants of Native American race/ethnicity (n = 12) were not included because none were infected with a Beijing strain; 2 Native American participants had disease that relapsed. CI, confidence interval.

We further evaluated the association between relapse and active disease caused by a Beijing strain among race/ethnicity groups by using stratified analysis. In an analysis limited to Asian–Pacific Islanders, adjustment for other risk factors for relapse had little effect on the association between relapse and active disease caused by a Beijing strain (adjusted OR 15.8, 95% CI 1.3–192, p = 0.03) (Table 5). Among other race/ethnicity groups, there was no association between relapse and active disease caused by a Beijing strain (Table 5).

**Table 5:** Multivariate analyses of association between active disease caused by a Beijing strain of *Mycobacterium* *tuberculosis* and relapse among race/ethnicity groups while controlling for other risk factors for relapse, Tuberculosis Trials Consortium Study 22*

Characteristic	Asian–Pacific Islander (n = 50)			Non-Hispanic black (n = 148)			Non-Hispanic white (n = 63)			Hispanic (n = 79)	
	OR (95% CI)	p value		OR (95% CI)	p value		OR (95% CI)	p value		OR (95% CI)	p value
Infected with Beijing strain	15.8 (1.3–192)	0.03		1.8 (0.5–6.5)	0.35		1.0 (0.1–7.7)	0.98		1.0 (0.1–13)	0.97
Underweight at tuberculosis diagnosis	3.1 (0.3–34)	0.35		2.9 (0.8–6.3)	0.15		11 (2.4–48)	<0.01		4.6 (0.9–24)	0.07
Pulmonary cavitation	2.1 (0.1–33)	0.60		4.0 (0.8–19)	0.09		2.7 (0.5–15)	0.25		6.6 (0.7–61)	0.09
Bilateral pulmonary disease	5.2 (0.4–69)	0.21		1.6 (0.5–4.8)	0.44		1.2 (0.2–9.9)	0.84		2.1 (0.3–15)	0.46
Two-month sputum culture positivity†	–	–		3.3 (1.1–9.7)	0.03		3.5 (0.6–20)	0.16		4.6 (0.5–40)	0.17

*The Tuberculosis Trials Consortium Study enrolled patients during 1995–1998. Participants in the case–control study were selected from among 1,004 HIV-infected participants. OR, odds ratio; CI, confidence interval. †Not included for Asian–Pacific Islander patients because none were culture positive at 2 months.

## Discussion

Using isolates from participants in a large prospective trial of supervised intermittent treatment, we found that active disease caused by a Beijing strain of *M*. *tuberculosis* was associated with a 2-fold increased risk for adverse TB treatment outcomes. The relationship between Beijing strains and treatment outcomes was driven by relapse because of their greater frequency and an apparent lack of any association between Beijing strains and treatment failure. Adjustment for the clinical, radiographic, and microbiologic risk factors for treatment failure and relapse and for treatment assignment in TBTC Study 22 had little effect on the association between active disease caused by a Beijing strain and the risk for relapse. In an exploratory analysis of the relationships of the effect of race/ethnicity on the association between Beijing strains and treatment outcomes, Asian–Pacific Islander patients were at increased risk for relapse if they had active disease caused by a Beijing strain, albeit with wide CIs around the risk estimate (adjusted OR 13.9, 95% CI 1.3–164).

Two other studies have evaluated the association between the Beijing genotype and relapse. A case–control analysis from Vietnam reported an adjusted OR of 3.2 for treatment failure or relapse among patients with active disease caused by a Beijing strain (*16*), and a cohort study from Singapore found an adjusted OR of 2.64 for relapse associated with Beijing strains (*17*). Our study provides additional support for the hypothesis that active disease caused by a Beijing strain is associated with increased risk for relapse (adjusted OR 2.2, 95% CI 1.0–4.9). The use of a nested case–control analysis of carefully characterized patients in a clinical trial enabled us to evaluate the relationship between Beijing strains and treatment outcomes in analyses adjusted for host factors previously associated with an increased risk for relapse. We were also able to eliminate effects of other possible confounding factors, such as treatment duration and adherence to treatment. Additionally, because reinfection could obscure the relationship between infection with a Beijing strain and the risk for relapse, we were able to remove cases of reinfection from this analysis. That adjustment for other risk factors for relapse, such as pulmonary cavitation and being underweight, had little effect on the association is additional evidence that active disease caused by a Beijing strain increases the risk for relapse.

After evolving in eastern Asia, the Beijing family of *M*. *tuberculosis* has spread around the world (*26*,*29*). Why might active disease caused by a Beijing strain confer an increased risk for relapse? Laboratory studies have suggested that Beijing strains may be better adapted for intracellular growth (*30*,*31*) and are more virulent in animal models of TB, perhaps by evading immune responses (*32*,*33*). Furthermore, an altered DNA repair enzyme in Beijing isolates that confers a mutator phenotype may confer greater flexibility to respond to adverse conditions (*34*), such as those posed by multidrug therapy.

The possibility that Beijing strains evade immune responses and are more virulent is generally borne out in studies with humans. The Beijing family is associated with extrapulmonary TB (*35*,*36*). That Beijing strains have been implicated in many outbreaks of TB suggests that they may be more efficiently transmitted or have an enhanced ability to progress to active disease than do other strains. In a study in Russia, active disease caused by a Beijing strain was associated with more severe radiographic manifestations of pulmonary TB (*11*). However, 2 smaller studies did not find an association between Beijing strains and radiographic severity of pulmonary TB (*37*,*38*). Because of its case–control design, our study cannot directly address the unresolved question of whether active disease caused by a Beijing strain is associated with radiographic severity of disease (such a comparison would require a cohort study design). However, adjustment for cavitation and bilateral pulmonary involvement did not affect the association between Beijing strains and relapse in our study. This finding suggests that the mechanism of the association between Beijing strains and relapse is not mediated by radiographic severity of disease.

Our study suggests that the increased risk for relapse associated with active disease caused by a Beijing strain may be related to the race/ethnicity of the patient; risk for relapse was higher for persons of Asian–Pacific Islander descent. There was also a trend toward increased risk for relapse among black patients infected with a Beijing strain, according to univariate analysis (OR 2.9, 95% CI 1.0–8.5; p = 0.07), although this trend was not retained in adjusted analysis (Table 5). It is notable that the population in which the Beijing genotype conferred the greatest risk for relapse was of Asian–Pacific Islander race/ethnicity and that the Beijing genotype evolved in eastern Asia. Coevolution of the Beijing genotype among persons of East Asian descent may have selected factors that contribute to transmissibility and a decreased response to therapy.

Our study has at least 4 limitations. First, the cohort from which this nested case–control analysis was drawn was composed of patients who enrolled in a randomized trial, who differed from the broader patient population at study sites. One clear bias in the study population is that TBTC Study 22 was limited to patients with drug-susceptible TB. Therefore, we cannot evaluate the association between Beijing strains and drug resistance. Second, despite the size of the study cohort, our case–control analysis had limited statistical power to detect associations, particularly in exploratory analyses of the relationships between *M*. *tuberculosis* genotype, race/ethnicity of the host, and the risk for relapse. Third, race/ethnicity was defined by using broad categories developed for the United States census and used in the TB surveillance system. However, these categories are crude approximations of the genetic background of patients. For example, the category Asian–Pacific Islander includes several distinct ethnic groups that have substantial differences in genetic backgrounds. We did not have access to human genetic material that would enable precise delineation of the genetic backgrounds of the trial’s participants. Fourth, we did not adjust for multiple comparisons, and our study should be viewed as an exploratory, hypothesis-generating analysis.

In summary, our study offers additional evidence that a common genotype of *M*. *tuberculosis*, the Beijing family, is associated with increased risk for relapse after completion of supervised short-course TB treatment. The finding that the population at greatest risk for relapse if they had active disease caused by a Beijing genotype was persons of Asian–Pacific Islander race/ethnicity suggests that the coevolution of this bacterial strain and the human population may have selected factors that confer a poor response to therapy.
